# Recombinant expression of natural *Arabidopsis* PRLIP1 variants reveals temperature-dependent differences in fluorescence and functional protein recovery in *Escherichia coli*

**DOI:** 10.1007/s11274-026-05138-y

**Published:** 2026-07-10

**Authors:** Gyöngyi Major, Sándor Kovács, Gábor Jakab

**Affiliations:** Department of Plant Biology, Institute of Biology, University of Pécs, Ifjúság útja 6, Pécs, H-7624 Hungary

**Keywords:** *Escherichia coli*, Esterase activity, Green fluorescent protein, Inclusion bodies, Natural allelic variation, Recombinant protein expression

## Abstract

Recombinant protein production in *Escherichia coli* is frequently limited by aggregation into inclusion bodies, reducing the recovery of active protein. In this study, we compared two naturally occurring *Arabidopsis thaliana* PRLIP1 variants from the Columbia-0 and Wassilewskija accessions, which differ by 13 amino acids, during temperature-controlled heterologous expression as GFP fusion proteins. GFP fluorescence, protein distribution between soluble and insoluble fractions, and esterase activity recovered after alkaline solubilization and refolding were analysed. Reduced cultivation temperatures increased GFP fluorescence and recovered esterase activity, although most of the recombinant protein still remained associated with inclusion bodies. Differences between the two PRLIP1 variants were observed in fluorescence intensity, inclusion body accumulation, and recovered esterase activity, indicating that natural PRLIP1 variation is associated with altered recombinant protein behaviour. Sequence-based hydropathy analysis revealed no major global differences in overall hydrophobicity, whereas aggregation prediction indicated subtle local differences in aggregation-prone regions. These results suggest that naturally occurring allelic variation can provide a useful first-pass approach to identify sequence-associated differences in folding-related behaviour and functional recovery, while residue-level causal mechanisms require further mutational analysis.

**Supplementary Information:**

The online version contains supplementary material available at 10.1007/s11274-026-05138-y.

## Introduction

*Escherichia coli* (*E. coli*) is still one of the most popular hosts for recombinant protein expression due to its well-characterized genetics, rapid growth, and extensive molecular tools designed for heterologous expression (Rosano et al. 2019). Despite the simple steps of recombinant expression - cloning of the target gene, induction, and purification - efficient recovery of soluble, functional protein is difficult because of protein misfolding, aggregation, and inclusion body (IB) formation (Kaur et al. 2018).

In *E. coli*, high-level expression of heterologous proteins often results in aggregation into IBs (Baig et al. 2014; Upadhyay et al. 2016). These aggregates are traditionally regarded as biologically inactive clusters of misfolded protein (Ramón et al. 2014). Recent studies indicate that IBs may contain proteins covering a range of conformational states, including partially folded and native-like species (Wang 2009; De Marco et al. 2019). In certain cases, enzymatic or fluorescent activity can be retained within IBs; therefore, aggregated proteins may preserve part of their native structure (García-Fruitós et al. 2005; Ventura and Villaverde 2006).

The structural and functional properties of IBs are affected by both intrinsic sequence determinants and expression conditions (Jevševar et al. 2005). Strategies such as co-expression of molecular chaperones (Mamipour et al. 2017), reduction of induction temperature (de Groot and Ventura 2006), earlier induction during exponential growth (San-Miguel et al. 2013), and optimization of inducer concentration (Mühlmann et al. 2017) have all been used to reduce aggregation and improve soluble protein yield. Newer approaches include supplementation with metabolic precursors or the use of co-culture systems to optimize host physiology (Slouka et al. 2019; Kumar et al. 2020; Chiang et al. 2020). Moreover, the metabolic burden of overexpression can be alleviated by tuning plasmid copy number and promoter strength (Lozano Terol et al. 2021).

In addition, rational protein engineering approaches aim to reduce aggregation propensity by identifying and modifying aggregation-prone sequence segments (van der Kant et al. 2019). Another widely used approach to increase recombinant protein yield involves site-directed mutagenesis to create rationally designed variants of the target protein (Chen et al. 2013). There is a link between intrinsic sequence properties and environmental factors that determines the behaviour of recombinant proteins. Recently, AI-assisted design has been successfully used for significantly enhancing soluble productivity of several proteins and enzymes mutants in *E. coli* (Jin et al. 2025; Khan and Khan 2026), but these molecules should be synthesized, which is time-consuming; for this reason, the natural allelic variants of the protein are good choices.

Promoter architecture and fusion partners additionally affect expression kinetics and folding outcomes. The arabinose-inducible pBAD system enables tight transcriptional control with minimal basal expression (Guzman et al. 1995; Schleif 2010). Green fluorescent protein (GFP), as a fusion tag, is frequently used to increase solubility and facilitate monitoring of folding efficiency, because its chromophore formation depends on correct folding of the β-barrel structure (Ormö et al. 1996). Consequently, fluorescence intensity provides a direct readout of conformational integrity and has been widely used as a folding reporter in recombinant systems (Drew et al. 2001; Bakholdina et al. 2021).

The PRLIP gene family encodes class 3 lipase-like proteins originally identified in *Arabidopsis thaliana* (Jakab et al. 2003). The recombinant expression of the PRLIP1 protein in bacteria and, therefore, its subsequent analysis, were insufficient due to extensive inclusion body formation. In the present study, we investigated the recombinant expression of PRLIP1 as a C-terminal GFP fusion in *E. coli* using a cycle 3 GFP variant adapted for bacterial expression (Crameri et al. 1996) and the tightly regulated pBAD promoter system. Because previous attempts to express PRLIP1 resulted predominantly in inclusion body formation and reduced activity (Jakab et al. 2003), we examined whether natural sequence variation between the Columbia-0 (Col-0) and Wassilewskija (Ws) accessions—differing by 13 amino acids, of which 7 are conserved substitutions with similar biochemical activities (SI.1b)—modulates folding efficiency and functional recovery. We further assessed the influence of cultivation temperature on solubility and enzymatic performance and tested whether GFP fluorescence quantitatively reflects the catalytic activity of the fusion protein. We combined sequence-based analysis with biochemical characterization. This study evaluates the impact of intrinsic sequence variation on recombinant protein behaviour and assesses the reliability of GFP fluorescence as an indicator of functional enzyme recovery under different expression conditions.

## Materials and methods

### Materials and reagents

All chemicals and reagents were purchased from Merck, unless specified. Restriction enzymes were obtained from Thermo Scientific™.

### Bacterial strains and media

*E. coli* TOP10 (Invitrogen) was used for cloning and expression of constructs. Transformants were selected on Luria-Bertani (LB; Duchefa Biochimie) agar plates or in LB broth supplemented with 100 µg/mL ampicillin (Duchefa Biochimie). The same *E. coli* strain was used for protein expression. It was maintained in LB medium supplemented with 0.02% L-arabinose for protein induction and 100 µg/mL ampicillin. The bacterial culture was performed at 37 °C for 4 h. After induction of protein expression, the growth temperature was reduced to 22 °C and 15 °C for 48 h, respectively.

### Construction of pBAD-GFP-PRLIP1 vectors

We fused the PRLIP1 protein to the C-terminal end of the GFP to monitor the folding and enhance the solubility of the recombinant protein (SI.2) (Hammarström et al. 2006). A linker region was used to fuse the C-terminal of GFP and the N-terminal of PRLIP1 (SI.1a). For heterologous expression of the GFP-PRLIP1 fusion protein in *E. coli*, the pBAD vector was used with an arabinose-inducible operon. The GFP optimized for the bacterium as a reporter protein (pBAD-GFP Cycle 3 Mutant, Maxygen) was used. The full-length coding region of PRLIP1 cDNA was obtained from the previous pGEX construct (Jakab et al. 2003). The fragments were inserted at the BamHI/KpnI site of the vector, and *E. coli* TOP10 cells were transformed with them. The constructs were verified (primer list in SI Table 1) by sequencing (Biocenter, Szeged, Hungary). Two types of PRLIP1 sequences were used: one representing the Columbia-0 (Col-0) accession of *Arabidopsis thaliana* and the other the Wassilewskija (Ws) accession. The PRLIP1 protein consists of 350 amino acids; the two sequences differ in 13 amino acids (SI.1b).

### GFP fluorescence

For whole-cell fluorescence measurements, GFP fluorescence spectra were recorded using a spectrofluorometer (RF-6000, Shimadzu, Japan). Medium log-phase cultures were centrifuged (13,500 rpm, 5 min, room temperature). The pellets were resuspended, and their cell numbers were determined at OD_600_ using a spectrophotometer (UV-1800, Shimadzu, Japan) and were diluted with buffer (10 mM Tris, pH 7.5, 0.15 M NaCl) to OD_600_ = 0.6, and then GFP fluorescence was measured in quartz cuvettes with an optical path length of 1 cm. The excitation and emission slit widths were set at 5 and 3 nm. The scanning speed was 600 nm/min, and the data were recorded every 2 nm. The excitation spectra were measured between 270 and 490 nm with an emission of 508 nm. The emission spectrum was measured between 410 and 700 nm with excitation at 390 nm. All measurements were performed in triplicate. The fluorescence matrix was excited between 300 and 450 nm, and the emissions were monitored between 460 and 590 nm at 1 nm intervals. The data were recorded using the Lab Solutions RF program.

### Growth assays on different substrates

Bacteria were grown on M9 basal salt medium (Sambrook et al. 1989) supplemented with 0.02% arabinose, 100 µg/mL ampicillin, and either 0.2% (w/v) of Tween20 or Glucose. The growth rates of the bacterial suspensions were determined by measuring the optical density (OD) at 600 nm using a spectrophotometer (UV-1800, Shimadzu, Japan).

### Protein expression of GFP and GFP-PRLIP1

*E. coli* TOP10 cells were cultivated in the presence of 100 µg/mL ampicillin. A single colony from cells transformed with pBAD-GFP was used to inoculate 3 mL of LB medium supplemented with ampicillin (100 µg/mL), and the culture was incubated overnight at 37 °C and 180 rpm. The overnight culture (2 mL) was used to inoculate 100 mL of LB medium supplemented with ampicillin (100 µg/mL), and the mixture was incubated under the conditions mentioned above. At an optical density of 0.4 at 600 nm, GFP expression was induced by the addition of 0.02% of L-arabinose, and cells were further incubated overnight at 37 °C. Expression of GFP was also optimized as a function of L-arabinose concentration. This test was performed according to the manufacturer’s instructions (Invitrogen) at final arabinose concentrations of 0.00002, 0.0002, 0.002, 0.02, and 0%. A final concentration of 0.02% proved to be the most effective; therefore, we consistently induced protein production with this concentration.

Based on literature data, the solubility of a protein produced by *E. coli* depends on the culture temperature (de Groot and Ventura 2006; Remington 2011; San-Miguel et al. 2013). Protein production was induced with 0.02% arabinose, and the cultures were grown at 22 °C and 15 °C, respectively, for an additional 48 h. The cells were then centrifuged (13,000 rpm, 5 min, RT) and the pellets were fresh-frozen at -80 °C until further use.

### Protein purification and refolding

The pellets were resuspended in 8 mL of potassium-phosphate buffer (prepared by dissolving 5 g of KH_2_PO_4_ and 10 g of K_2_HPO_4_ in 1000 mL of distilled water). The resuspended cells were stored at -80 °C for 24 h. The cells were mechanically disrupted using a French press (PC-160, SLM Aminco) at 4000–12,000 psig, operated by a Wabash hydraulic press (Wabash, Indiana), and subjected to a sudden release. The lysed cells were then further processed by alternative methods. Following centrifugation (13000 rpm, 5 min, 4 °C), the supernatant (soluble proteins) and the pellet (inclusion bodies) were analysed by SDS-PAGE.

The solubilization of inclusion bodies is based on the method described by Singh (Singh et al. 2008) with a few modifications. It was performed at a high pH (12.0) and a low urea concentration (2 M). Briefly: 1 mL of bacterial culture was centrifuged (3 min, 13,000 rpm, RT). The pellet was resuspended in 100 µL of distilled water. After the addition of 1000 µL solubilization buffer (Tris-HCl 100 mM/L pH 12.0, 2 M/L urea) and incubation at RT for 30 min, the samples were centrifuged (13,000 rpm, 20 min, 4 °C). The pH was restored: to the supernatant (approximately 1 mL), 9 mL of refolding buffer (Tris-HCl, 50 mM/L pH 8.5, sucrose 5% w/v, urea 2 M/L) was added at a rate of 10 µL/min by pulsatile dilution. The samples were incubated at 4 °C for 2 h and then centrifuged (15000 rpm, 30 min, 4 °C). Further measurements were done with the supernatants.

### SDS-PAGE

Recombinant proteins were separated by electrophoresis using a 5% (w/v) stacking gel and a 13% (w/v) resolving gel in a Mini-Protean II electrophoresis cell (Bio-Rad Laboratories, Richmond, CA) under the standard denaturing conditions according to the method of Laemmli (Laemmli 1970) with prior heating of the samples (5 min at 95 °C) in a buffer containing 2% SDS. The run was carried out at a constant voltage of 150 V. Proteins were detected in gels by Coomassie Brilliant Blue R-250 staining, and their molecular masses were determined by referring to the mobility of known molecular mass standards (PageRuler™ Unstained Protein Ladder, Thermo Scientific™).

### In-gel fluorescence reading

Samples for SDS-PAGE were mixed in a 1:1 ratio with loading buffer (100 mM Tris-HCl, pH 6.8, 2% w/v SDS, 0.2% w/v bromophenol blue, 10% v/v β-mercaptoethanol, and 20% v/v glycerol) and loaded directly onto the gel without heating. Following SDS-PAGE, a fluorescent image of GFP-PRLIP1 on the gel was captured using the UVP Benchtop UV Transilluminator (Daigger Scientific Inc.). The GFP bands were visible in the gel under UV illumination, and no additional bands were observed.

### Esterase assay

The enzymatic esterase activity of recombinant GFP-PRLIP1 fusion proteins was spectrophotometrically assayed using p-nitrophenyl butyrate (p-NPB) as substrate. After protein expression, soluble and insoluble fractions were separated by centrifugation (13,000 rpm, 5 min, 4 °C). The insoluble inclusion body-derived fractions were solubilized and refolded as described above, and the resulting refolded protein preparations were used for activity measurements. The total protein assay was performed as described by Bradford (Bradford 1976) with bovine serum albumin as a standard. The refolded enzyme solutions obtained at different culture temperatures were normalized for protein content prior to activity measurements. The reaction mixture was freshly prepared by mixing 1 volume of a 10 mM p-NPB dissolved in methanol with 18 volumes of 10 mM HEPES buffer, pH 6.5. For each assay, 40 µL of the enzyme extract and 760 µL of the reaction mixture were placed in a narrow cuvette. The release of 4-nitrophenolate was monitored at 415 nm at 37 °C for 15 min at 1-min intervals against a blank containing reaction mixture without enzyme preparation. Product formation was calculated using a molar extinction coefficient of 14,000 M⁻¹ cm⁻¹ for 4-nitrophenolate at 415 nm (Shirai and Jackson 1982). Only the linear phase of the reaction was used for activity calculations. The initial reaction rate was determined from the slope of the absorbance increase at 415 nm. Esterase activity was calculated from the rate of 4-nitrophenolate formation and normalized to the amount of total protein present in the assay. Specific esterase activity was expressed as nU µg⁻¹ total protein. All assays were performed in triplicate, and values are presented as means.

For relative correction of esterase activity according to GFP-PRLIP1 target protein abundance, GFP-PRLIP1 bands were quantified from Coomassie-stained SDS-PAGE gels by densitometric analysis. Equal total protein amounts were loaded in each lane, and band intensities were background-corrected before analysis. For each variant, GFP-PRLIP1 band intensities in the inclusion body fraction were normalized to the corresponding 37 °C inclusion body sample, which was set to 1.00. Relative target-abundance-corrected recovered activity was calculated by dividing the total-protein-normalized esterase activity by the relative GFP-PRLIP1 band abundance. Because this correction was based on relative densitometric values rather than absolute target protein quantification, the corrected values were interpreted as relative recovered activity corrected for GFP-PRLIP1 abundance and not as absolute catalytic efficiency.

### Sequence-based computational analyses

Hydropathy profiles of naturally occurring PRLIP1 variants were generated using the Kyte–Doolittle scale with a nine-residue sliding window implemented in Python. Intrinsic β-aggregation propensity was predicted using the TANGO algorithm (Fernandez-Escamilla et al. 2004) under standard conditions (pH 7.0, 298 K, ionic strength 0.15 M). N- and C-termini were considered unmodified. Per-residue aggregation scores and cumulative aggregation values were calculated for each accession-specific PRLIP1 variants using identical parameters to allow relative comparison of predicted aggregation-prone regions.

### Statistical analysis

Each treatment group consisted of three bacterial strains. All measurements were performed in three to four repetitions. Results are represented as means ± standard deviations. The effect of culture temperature on the different vector constructions was analysed with a two-way ANOVA. Three null hypotheses were tested: (1) the culture temperature had no effect on the bacterial growth, (2) fusion of PRLIP1 had no effect on the bacterial growth, and (3) there was no interaction between the two factors. Tukey HSD was used as a post-hoc test, and verified rejections of the ANOVA null hypotheses were characterised by p-values. Pearson correlation coefficients were calculated in PAST, and statistical significance was evaluated at *p* < 0.05. Statistical analyses were performed using the PAST software (Hammer et al. 2001).

## Results

### Sequence-based comparison of PRLIP1 variants

To determine if intrinsic sequence features contribute to the differences observed between PRLIP1 variants, we conducted a comparative hydropathy analysis of the Col-0 and Ws amino acid sequences (SI.3). Using a sliding-window Kyte–Doolittle profile (window size = 9 residues), we found highly similar global hydrophobicity patterns across the 350-amino-acid sequence. The 13 polymorphic residues are distributed throughout the protein and do not generate new significant hydrophobic segments. Overall, no major differences in hydrophobicity were observed between the two naturally occurring PRLIP1 variants. To further examine the potential variation in β-aggregation propensity, TANGO analysis was performed under standard conditions (pH 7.0, 298 K, ionic strength 0.15 M). Both PRLIP1 variants showed overlapping aggregation profiles, with three key aggregation-prone regions around residues 33–45, 85–92, and 159–164 (SI.4). The Col-0 variant had a modestly higher total aggregation score (1077.69) than the Ws variant (1003.10), corresponding to an approximately 7% increase in predicted aggregation tendency. Polymorphic residue 38 lies within the N-terminal aggregation hotspot, and residue 84 is next to the second aggregation-prone region. Localized sequence variation may therefore influence aggregation behaviour without changing the overall aggregation landscape.

### Production of recombinant GFP-PRLIP1 fusion proteins in *E. coli*

**Fig. 1:**
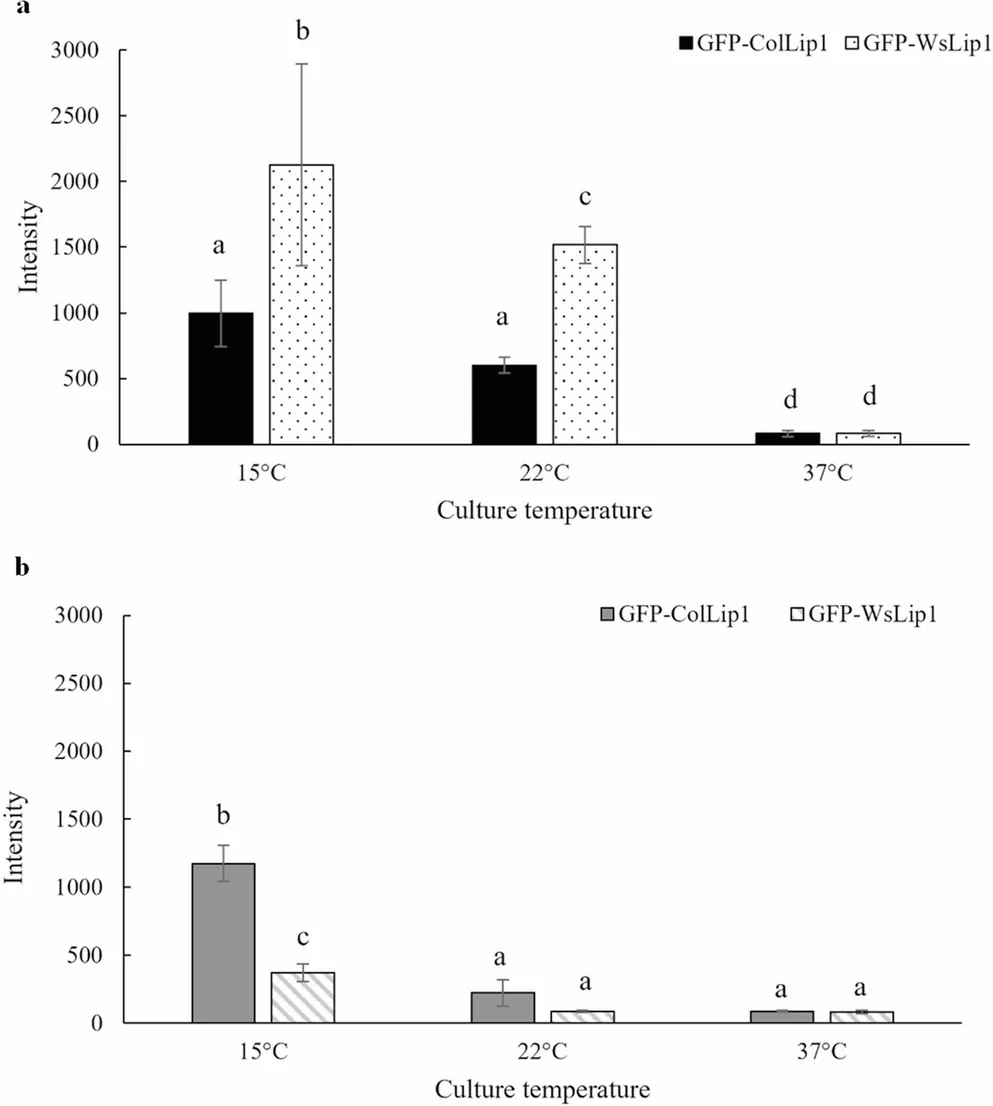
GFP fluorescence of PRLIP1 fusion proteins at different temperatures. **a** Fluorescence intensity of inclusion bodies from strains expressing GFP-ColLip1 or GFP-WsLip1 grown at 37 °C, 22 °C, or 15 °C. **b** Fluorescence intensity of the corresponding supernatant fractions. Lowercase letters above the bars indicate statistically significant differences between the analysed groups, as determined by two-way ANOVA performed in Past (*n* = 6)

Recombinant expression of GFP and GFP-PRLIP1 fusion constructs was successfully in *Escherichia coli* TOP10 cells with an arabinose-inducible operon (SI.5). Fluorescence emission spectra of all constructs exhibited the characteristic GFP excitation and emission maxima (excitation at 395 nm, emission at 508 nm), confirming correct chromophore formation in expressed proteins (SI.6). Whole-cell fluorescence measurements were performed to evaluate temperature-dependent expression efficiency. Cells expressing GFP alone displayed strong fluorescence at all tested temperatures (37 °C, 22 °C, and 15 °C). In contrast, fluorescence intensity of the GFP-PRLIP1 fusion constructs was highly reduced at 37 °C. Cultivation at lower temperatures (22 °C and 15 °C) resulted in significantly increased fluorescence intensity for both fusion variants (Fig. 1a, b). For the GFP-only construct, fluorescence was highest at 22 °C and 37 °C and moderately reduced at 15 °C. In comparison, both GFP-PRLIP1 variants exhibited maximal fluorescence at reduced temperatures, with measurable differences between the Col-0 (GFP-ColLip1) and Ws (GFP-WsLip1) constructs. Despite temperature-dependent enhancement, fluorescence intensity of the fusion proteins remained lower than that of GFP expressed alone under identical conditions (SI.7). Cultivation temperature influences the accumulation of fluorescent GFP-PRLIP1 protein, and natural sequence variation between the two PRLIP1 variants affects fluorescence yield in the heterologous expression system.

### Effect of temperature on the production of inclusion bodies

**Fig. 2:**
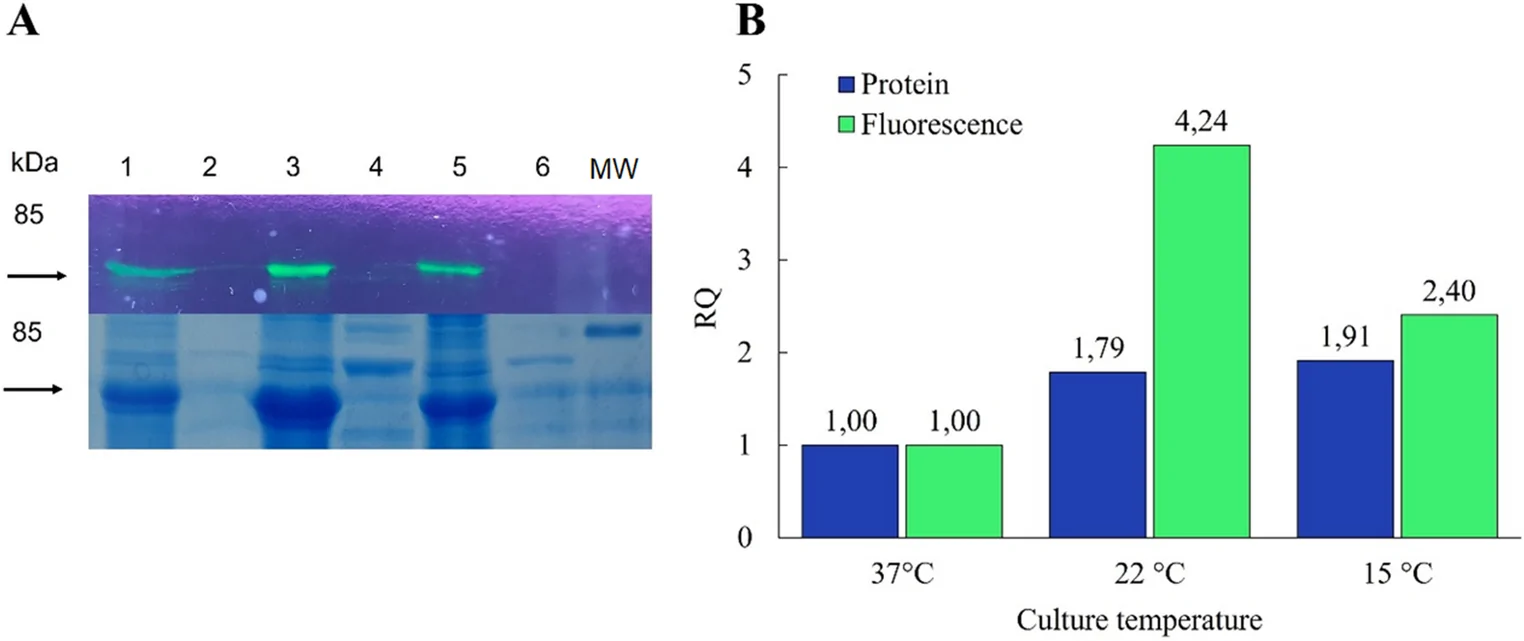
Analysis of recombinant GFP-PRLIP1 (WsLip1) protein expression and fluorescence under different culture temperatures. **a** SDS-PAGE analysis of *E. coli* expressing pBAD-GFP-WsLip1, showing in-gel GFP fluorescence and Coomassie Brilliant Blue staining. The GFP-PRLIP1 fusion protein (~ 64 kDa) is indicated by arrows. Lane 1: inclusion body (37 °C); Lane 2: supernatant (37 °C); Lane 3: inclusion body (22 °C); Lane 4: supernatant (22 °C); Lane 5: inclusion body (15 °C); Lane 6: supernatant (15 °C); Lane 7: molecular weight marker (85 kDa is indicated). **b** Densitometric comparison of total GFP-PRLIP1 protein content (Coomassie) and GFP fluorescence in inclusion bodies across culture temperatures. GFP intensity and protein levels are expressed relative to the 37 °C condition (set as 1)

**Fig. 3:**
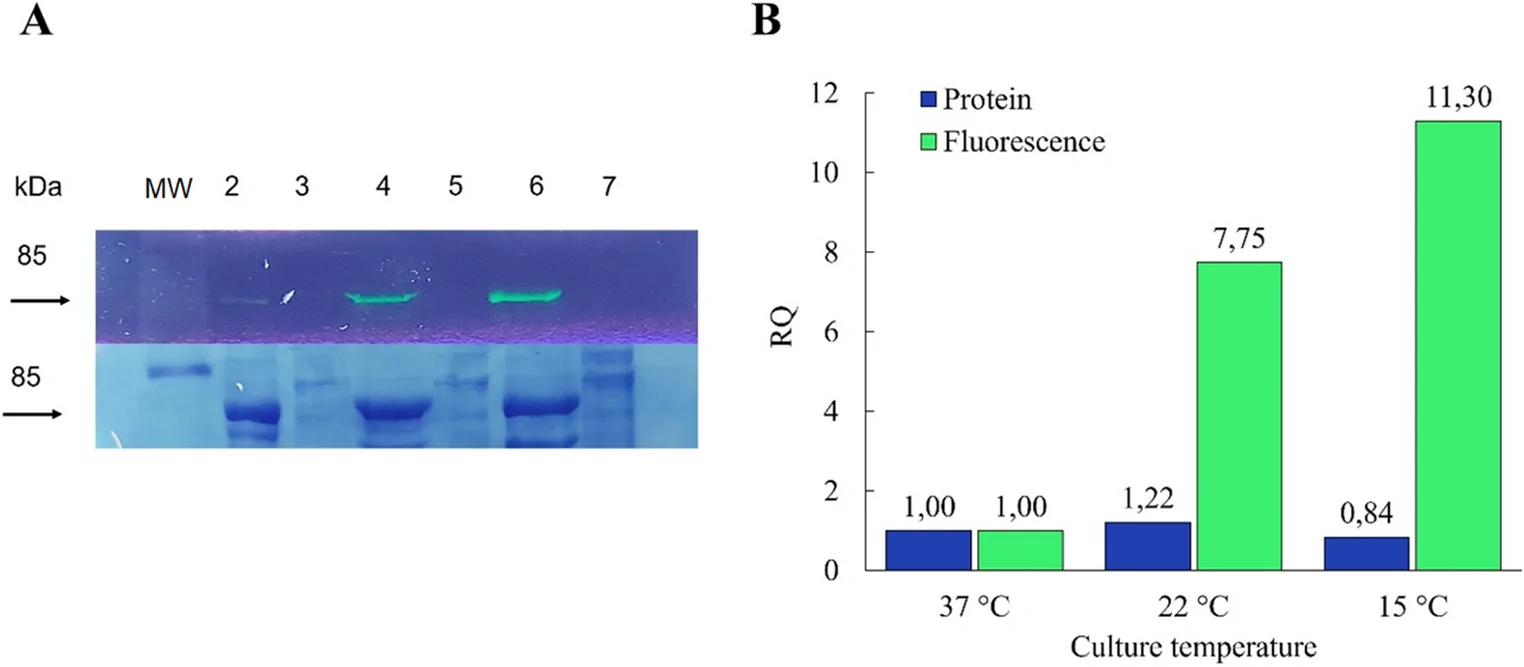
Analysis of recombinant GFP-PRLIP1 (ColLip1) protein expression and fluorescence under different culture temperatures. **a** SDS-PAGE analysis of *E. coli* expressing pBAD-GFP-ColLip1, showing in-gel GFP fluorescence and Coomassie Brilliant Blue staining. The GFP-PRLIP1 fusion protein (~ 64 kDa) is indicated by arrows. Lane 1: molecular weight marker (85 kDa is indicated); Lane 2: inclusion body (37 °C); Lane 3: supernatant (37 °C); Lane 4: inclusion body (22 °C); Lane 5: supernatant (22 °C); Lane 6: inclusion body (15 °C); Lane 7: supernatant (15 °C). **b** Densitometric comparison of total GFP-PRLIP1 protein content (Coomassie) and GFP fluorescence in inclusion bodies across culture temperatures. Values are normalized to the 37 °C condition (set as 1)

Expression of the GFP-PRLIP1 fusion constructs in *E. coli* produced a dominant band around 64 kDa, corresponding to the expected molecular weight of the fusion protein (Figs. 2a and 3a). After arabinose induction, the recombinant protein was visible across all tested cultivation temperatures (37 °C, 22 °C, 15 °C). Cell lysis and fractionation revealed that most of the recombinant protein was in the insoluble pellet fraction in all tested conditions. Densitometric analysis of Coomassie-stained gels showed higher accumulation in inclusion bodies than in the supernatant. When normalized to 37 °C, cultivation at lower temperatures increased total recombinant protein levels in both accession-specific PRLIP1 variants (Figs. 2b and 3b). In-gel fluorescence analysis confirmed GFP-PRLIP1 presence in both soluble and insoluble fractions (Figs. 2a and 3a). Fluorescence intensity in the inclusion bodies increased at lower cultivation temperatures, despite the dominance of insoluble protein. Although total recombinant protein did not scale proportionally, GFP fluorescence within the inclusion bodies increased at lower temperatures (Figs. 2a and 3a). A weaker, yet detectable fluorescent signal was observed in the supernatant, which does not represent host protein background, as control samples containing host proteins alone did not show a band at the corresponding molecular weight (data not shown). Construct-specific differences were also observed: the Col-0 construct had higher protein levels at 15 °C than at 22 °C, while the Ws construct accumulated more at 22 °C than at 15 °C. Despite these differences, both natural allelic variants of PRLIP1 showed increased recombinant protein production at reduced cultivation temperatures compared with 37 °C, with comparable total amounts at 22 °C.

### In vivo esterase activity of the recombinant fusion protein GFP-PRLIP1

**Fig. 4:**
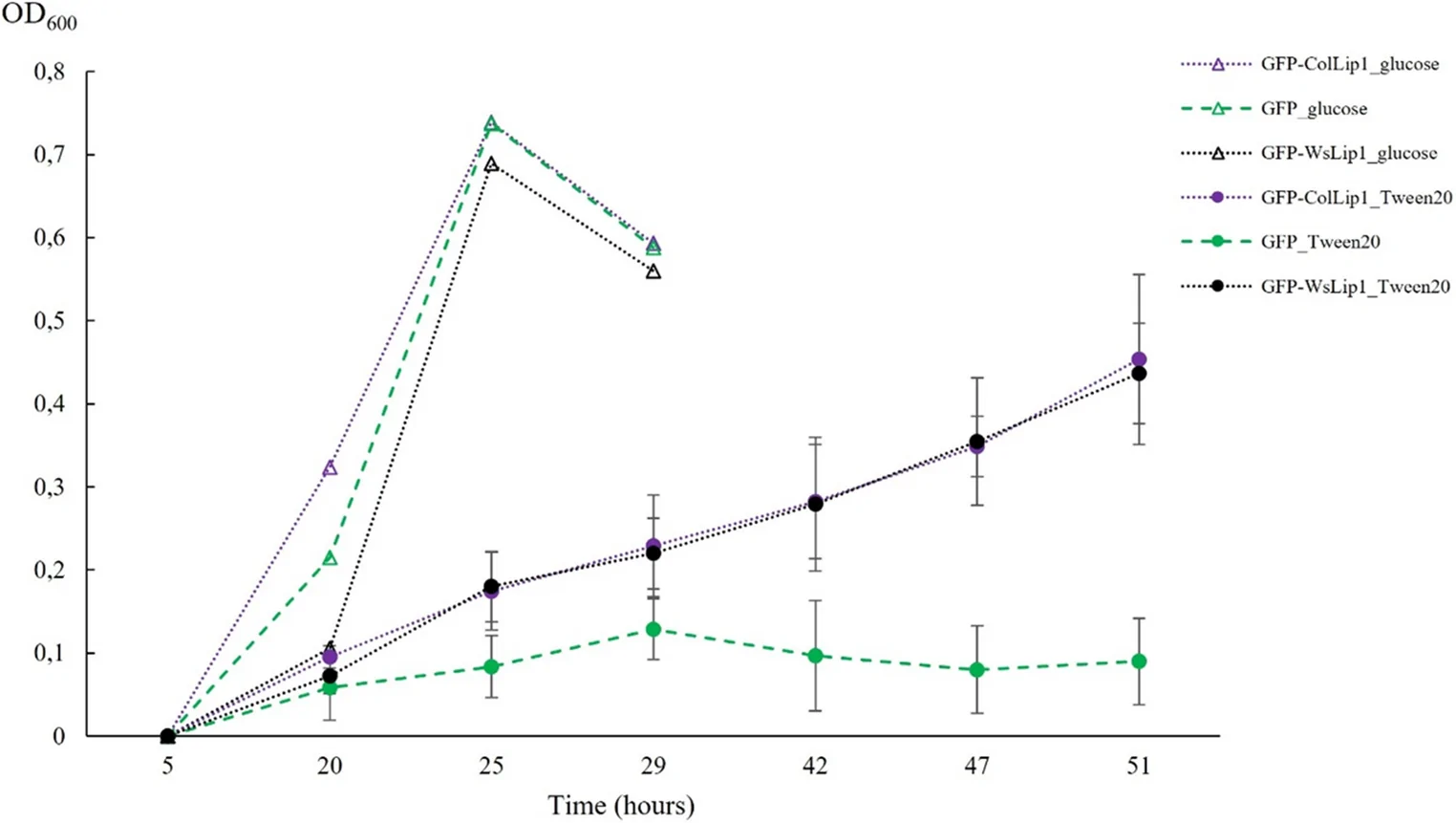
Heterologous expression and in situ assay of lipolytic acyl hydrolase activity of PRLIP1 in *E. coli* cultured in minimal medium. *E. coli* cells expressing the GFP-PRLIP1 fusion protein were grown in M9 basal salt medium supplemented with L-arabinose and either glucose or Tween20 as the sole carbon source. Bacterial growth was monitored by measuring the optical density at 600 nm (OD₆₀₀). The experiment was performed in three independent biological replicates with consistent results

To determine whether the recombinant fusion proteins retained their catalytic activity in vivo, transformed *E. coli* strains were cultivated at 22 °C in M9 minimal medium, supplemented with either glucose or Tween20 as the unique carbon source. Bacterial growth was monitored over 30–50 h under identical conditions by measuring optical density at 600 nm. All strains grew efficiently in glucose-containing medium, confirming similar general growth capacity during induction. In contrast, only strains expressing GFP-PRLIP1 could grow in medium with Tween20 as the only carbon source (Fig. 4). Control strains expressing GFP alone did not show measurable growth under these conditions. Both PRLIP1 variants supported growth in the presence of Tween20, indicating retained esterase activity in the heterologous host. The growth patterns of the Col-0 and Ws constructs were comparable, consistent with similar functional activity in vivo under the tested conditions. The GFP-PRLIP1 fusion proteins are catalytically active in *E. coli* and can hydrolyse Tween20 to support bacterial growth.

### In vitro esterase activity of the recombinant fusion protein GFP-PRLIP1

Esterase activity of the recombinant GFP-PRLIP1 fusion proteins was measured using p-nitrophenyl butyrate (p-NPB) as substrate. Inclusion body fractions were solubilized and refolded as described in Materials and Methods, with enzyme preparations normalized to total protein concentration before activity measurements to allow comparison of specific activity. Hydrolysis of p-NPB was monitored spectrophotometrically at 415 nm by tracking p-nitrophenolate release, and specific esterase activity was expressed as nU enzyme per µg total protein. Under all tested conditions, substrate conversion was linear within the measurement interval, and assays were performed under identical substrate concentrations. After alkaline solubilization and dilution-mediated refolding, GFP-PRLIP1 preparations showed detectable esterase activity, indicating recovery of catalytically competent protein from the inclusion body-derived fraction. Specific esterase activity increased as cultivation temperature decreased for both PRLIP1 variants (Table 1). Proteins expressed at 37 °C showed the lowest activity, while preparations obtained from cultures grown at 22 °C and 15 °C displayed significantly higher esterase activity. Variant-dependent differences were also observed: the Ws-derived fusion protein (GFP-WsLip1) showed higher specific activity than the Col-0 construct (GFP-ColLip1) at reduced cultivation temperatures, with comparable activities at 37 °C. Since enzyme preparations were normalized to protein concentration before testing, the observed differences should be interpreted as differences in recovered activity from total refolded protein preparations, rather than as intrinsic catalytic efficiency. These results underline that both cultivation temperature and PRLIP1 sequence variation influence the functional recovery of recombinant PRLIP1 after solubilization and refolding from inclusion body-derived fractions. To evaluate whether the temperature-dependent increase in esterase activity could be explained solely by differences in GFP-PRLIP1 target protein abundance, activity values were additionally corrected using the relative densitometric intensity of the GFP-PRLIP1 band in the corresponding inclusion body fraction. This analysis showed that recovered esterase activity remained higher at reduced cultivation temperatures after correction for relative GFP-PRLIP1 abundance (SI.Table 3). Thus, the increased activity observed at 22 °C and 15 °C cannot be attributed only to higher accumulation of the target fusion protein in the inclusion body fraction, but also reflects improved recovery of catalytically competent protein after solubilization and refolding.

**Table 1:** Esterase activity of GFP–PRLIP1 recovered from the insoluble fraction after alkaline solubilization and refolding

	GFP-WsLip1	GFP-ColLip1
Culture temperature	nU µg^− 1^ total protein	nU µg^− 1^ total protein
**37 °C**	0.155 ± 1.63E-07	0.208 ± 6.98E-08
**22 °C**	0.619 ± 1.53E-08	0.502 ± 2.37E-08
**15 °C**	1.218 ± 2.02E-08	0.869 ± 5.12E-08

Inclusion body-derived protein preparations obtained from cultures grown at 37, 22, and 15 °C were solubilized, refolded, and normalized to total protein content before activity measurements. Esterase activity was determined using p-nitrophenyl butyrate (p-NPB) as substrate by monitoring p-nitrophenolate formation at 415 nm. Activity values were calculated from the initial linear increase in absorbance and expressed as nU µg⁻¹ total protein. Values represent means ± SD of three independent measurements. Substrate conversion remained linear throughout the 15-min assay interval

### Correlation between fluorescence intensity, esterase activity, and cultivation temperature

**Fig. 5:**
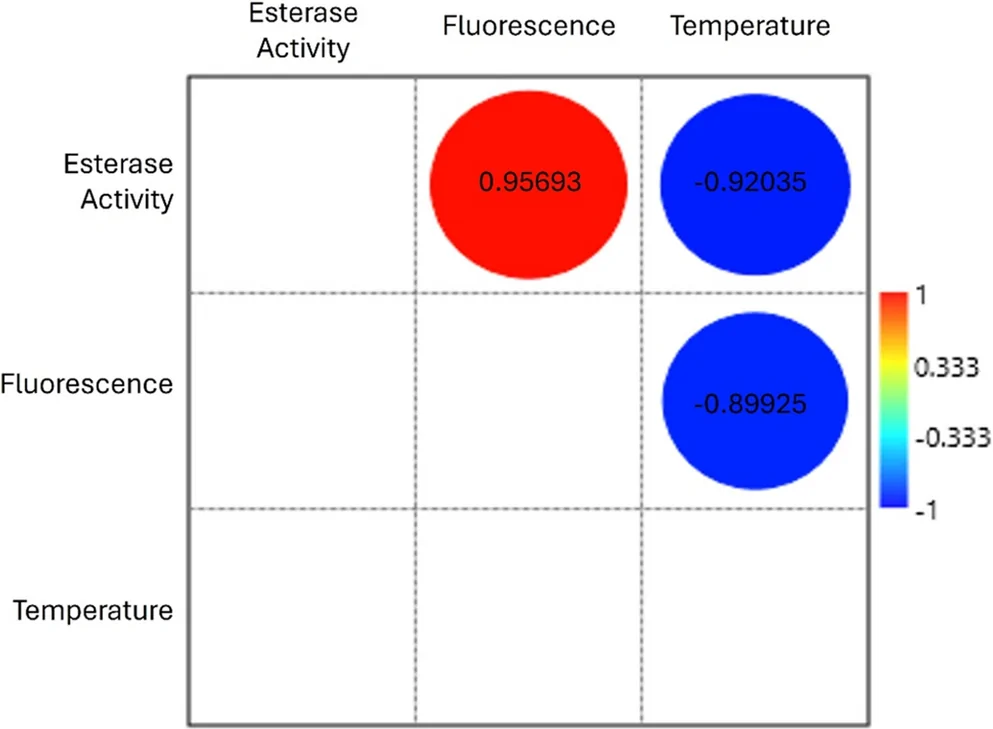
Correlation matrix of esterase activity, GFP fluorescence, and cultivation temperature. The matrix visualizes the relationship between culture temperature, GFP fluorescence intensity, and esterase activity. Circle colour and size represent the strength and direction of the correlation (scale shown on the right): red circles denote strong positive correlations, whereas blue circles indicate strong negative correlations. Numerical values inside each circle correspond to the Pearson correlation coefficients (r) for the respective variable pairs

To examine the relationship between GFP fluorescence and enzyme activity, Pearson correlation analysis was performed using fluorescence intensity, specific esterase activity, and cultivation temperature as variables (Fig. 5). In this pooled analysis, a strong positive correlation was observed between fluorescence intensity and specific esterase activity (*r* = 0.95693), indicating that increased fluorescence was associated with enhanced catalytic performance of the recombinant protein. In contrast, cultivation temperature showed a strong negative correlation with both fluorescence intensity (*r* = -0.89925) and specific esterase activity (*r* = -0.92035), indicating better functional recovery at reduced temperatures. Because cultivation temperature strongly influenced both fluorescence and enzymatic activity, we further analysed the fluorescence–activity relationship separately within each temperature condition. No correlation was observed at 37 °C (*r* = − 0.019, *p* = 0.958), whereas strong positive correlations were detected at 22 °C (*r* = 0.974, *p* = 1.86 × 10⁻⁶) and 15 °C (*r* = 0.726, *p* = 0.027) (SI.Table 2). These results indicate that GFP fluorescence reflects enzymatically active GFP–PRLIP1 under low-temperature expression conditions, particularly at 22 °C and 15 °C, but not at 37 °C. Thus, fluorescence intensity can be used as an indicator of functional protein recovery under permissive low-temperature cultivation conditions, while its predictive value is limited under high-temperature expression.

## Discussion

Recombinant expression of GFP-PRLIP1 fusion proteins in *Escherichia coli* indicated that minor natural sequence variation was associated with differences in folding-related behaviour and functional recovery in a heterologous host. The two accession-specific PRLIP1 variants, differing by 13 amino acids, showed measurable differences in fluorescence intensity, inclusion body formation, and specific esterase activity. These observations suggest that small intrinsic sequence differences may be associated with altered aggregation behaviour and the recovery of catalytically active protein during recombinant production.

The effect of cultivation temperature on recombinant protein solubility and activity observed in this study is in agreement with previous studies showing that reduced expression temperatures can enhance folding efficiency and functional yield in *E. coli* (Baneyx and Mujacic 2004; Sørensen and Mortensen 2005). Lower temperatures may slow translation rates and aggregation processes, promoting successful folding pathways. Despite these improvements, most of the GFP-PRLIP1 proteins were still localized in inclusion bodies, a common outcome for aggregation-prone proteins expressed at high levels (Carrió and Villaverde 2001). Our data indicate that lowering cultivation temperature improves functional recovery but does not necessarily prevent inclusion body formation.

It is important to distinguish between the presence of native-like protein species in inclusion bodies and the enzymatic activity measured after refolding. In the present study, esterase activity was not determined directly in intact inclusion bodies. Instead, the insoluble fraction was solubilized at pH 12.0 and subsequently refolded by dilution before activity measurement. Therefore, the detected esterase activity reflects the recovery of catalytically competent GFP-PRLIP1 after alkaline solubilization and refolding, rather than the intrinsic activity of the protein within intact inclusion bodies. The measured activity is therefore influenced by both the amount of recombinant protein present in the insoluble fraction and the efficiency with which the protein can be solubilized and refolded into an active conformation.

The use of GFP as a folding reporter is well established. Waldo et al. (Waldo et al. 1999) demonstrated that GFP fluorescence can indicate folding efficiency of fusion proteins, while later studies found that GFP may remain fluorescent even in inclusion bodies (García-Fruitós et al. 2007). Consistent with the data, we observed fluorescence in both soluble and insoluble GFP-PRLIP1 fractions. However, fluorescence should not be interpreted solely as a measure of protein accumulation. In our temperature-specific correlation analysis, GFP fluorescence strongly correlated with specific esterase activity at 22 °C and 15 °C, but not at 37 °C. These results indicate that fluorescence intensity reflects functional recovery of GFP-PRLIP1 under low-temperature expression conditions, whereas its predictive value is limited at 37 °C, where folding is likely impaired.

Sequence-dependent differences further support the idea that localized amino acid variation can influence recombinant protein behaviour. Protein engineering studies show that even single amino acid substitutions can strongly influence solubility and expression (Dale et al. 1994; van der Kant et al. 2019). Instead of using site-directed mutagenesis, which can negatively affect the enzyme activity of the protein, we rather took advantage of natural sequence variants. The natural polymorphisms between the Col-0 and Ws variants appear to affect folding behaviour similarly while maintaining the protein’s biological function. Hydropathy analysis based on sequence data did not reveal major overall differences, implying that localized effects, rather than changes in global hydrophobicity, explain the variations in activity and fluorescence. Computational aggregation prediction indicated highly similar global aggregation tendencies between the two accession-specific PRLIP1 variants. However, we could detect subtle quantitative differences, particularly in the region encompassing residues 85–92, where the Col-0 variant showed increased aggregation propensity. These localized sequence polymorphisms likely alter specific aggregation-prone segments by influencing folding efficiency and solubility without causing large-scale changes in hydrophobicity.

Both natural sequence polymorphism and cultivation temperature were associated with differences in the recovery of enzymatically active GFP-PRLIP1 after solubilization and refolding from inclusion body-derived fractions. Optimization of these parameters may therefore be important for improving functional recovery of aggregation-prone plant enzymes in heterologous expression systems. Although the present study does not resolve the contribution of individual amino-acid substitutions, it highlights the usefulness of naturally occurring variants as a practical approach to examine sequence-associated differences in recombinant protein behaviour. Instead of generating artificial point mutants, which is time-consuming and may disrupt the native sequence context and native function of the enzyme, naturally occurring variants allow comparison of alternative forms of the same protein that have been maintained in plant populations by natural selection. This approach cannot replace targeted mutagenesis when residue-level causal mechanisms are required, but it can serve as a cost-effective first-pass strategy to identify sequence variants associated with altered folding, aggregation behaviour, solubility, and functional recovery in heterologous expression systems. Such a strategy may be applicable not only to PRLIP1, but also to other plant proteins for which natural allelic variation is available.

## Supplementary Information

Below is the link to the electronic supplementary material.

Supplementary Material 1

## Data Availability

No datasets were generated or analysed during the current study.

## References

[CR1] Baig F, Fernando LP, Salazar MA et al (2014) Dynamic transcriptional response of *Escherichia coli* to inclusion body formation. Biotechnol Bioeng 111:980–999. 10.1002/bit.25169 24338599 10.1002/bit.25169 PMC3969792

[CR2] Bakholdina SI, Stenkova AM, Bystritskaya EP et al (2021) Studies on the structure and properties of membrane phospholipase A1 inclusion bodies formed at low growth temperatures using gfp fusion strategy. Molecules 26:3936. 10.3390/MOLECULES26133936 34203222 10.3390/molecules26133936 PMC8271855

[CR3] Baneyx F, Mujacic M (2004) Recombinant protein folding and misfolding in *Escherichia coli*. Nat Biotechnol 22:1399–1408. 10.1038/nbt1029 15529165 10.1038/nbt1029

[CR4] Bradford MM (1976) A rapid and sensitive method for the quantitation of microgram quantities of protein utilizing the principle of protein-dye binding. Anal Biochem 72:248–254. 10.1016/0003-2697(76)90527-3 942051 10.1016/0003-2697(76)90527-3

[CR5] Carrió MM, Villaverde A (2001) Protein aggregation as bacterial inclusion bodies is reversible. FEBS Lett 489:29–33. 10.1016/S0014-5793(01)02073-7 11231008 10.1016/s0014-5793(01)02073-7

[CR6] Chen S, Huang Z, Wu J et al (2013) Combination of site-directed mutagenesis and calcium ion addition for enhanced production of thermostable MBP-fused heparinase i in recombinant *Escherichia coli*. Appl Microbiol Biotechnol 97:2907–2916. 10.1007/s00253-012-4145-6 22588503 10.1007/s00253-012-4145-6

[CR7] Chiang C-J, Hu M-C, Chao Y-P (2020) A strategy to improve production of recombinant proteins in *Escherichia coli* based on a glucose-glycerol mixture and glutamate. J Agric Food Chem 68:8883–8889. 10.1021/ACS.JAFC.0C03671 32806130 10.1021/acs.jafc.0c03671

[CR8] Crameri A, Whitehorn EA, Tate E, Stemmer WPC (1996) Improved green fluorescent protein by molecular evolution using DNA shuffling. Nat Biotechnol 14:315–319. 10.1038/nbt0396-315 9630892 10.1038/nbt0396-315

[CR9] Dale GE, Broger C, Langen H et al (1994) Improving protein solubility through rationally designed amino acid replacements: solubilization of the trimethoprim-resistant type SI dihydrofolate reductase. Protein Eng 7:933–939. 10.1093/protein/7.7.933 7971955 10.1093/protein/7.7.933

[CR10] de Groot NS, Ventura S (2006) Effect of temperature on protein quality in bacterial inclusion bodies. FEBS Lett 580:6471–6476. 10.1016/j.febslet.2006.10.071 17101131 10.1016/j.febslet.2006.10.071

[CR11] De Marco A, Ferrer-Miralles N, Garcia-Fruitós E et al (2019) Bacterial inclusion bodies are industrially exploitable amyloids. FEMS Microbiol Rev 43:53–72. 10.1093/FEMSRE/FUY038 30357330 10.1093/femsre/fuy038

[CR12] Drew DE, Von Heijne G, Nordlund P, De Gier JWL (2001) Green fluorescent protein as an indicator to monitor membrane protein overexpression in *Escherichia coli*. FEBS Lett 507:220–224. 10.1016/S0014-5793(01)02980-5 11684102 10.1016/s0014-5793(01)02980-5

[CR13] Fernandez-Escamilla AM, Rousseau F, Schymkowitz J, Serrano L (2004) Prediction of sequence-dependent and mutational effects on the aggregation of peptides and proteins. Nat Biotechnol 22:1302–1306. 10.1038/nbt1012 15361882 10.1038/nbt1012

[CR15] García-Fruitós E, González-Montalbán N, Morell M et al (2005) Aggregation as bacterial inclusion bodies does not imply inactivation of enzymes and fluorescent proteins. Microb Cell Fact 4:1–6. 10.1186/1475-2859-4-27/FIGURES/3 16156893 10.1186/1475-2859-4-27 PMC1224866

[CR14] García-Fruitós E, Arís A, Villaverde A (2007) Localization of functional polypeptides in bacterial inclusion bodies. Appl Environ Microbiol 73:289–294. 10.1128/AEM.01952-06 17085715 10.1128/AEM.01952-06 PMC1797118

[CR16] Guzman LM, Belin D, Carson MJ, Beckwith J (1995) Tight regulation, modulation, and high-level expression by vectors containing the arabinose P(BAD) promoter. J Bacteriol 177:4121–4130. 10.1128/JB.177.14.4121-4130.1995 7608087 10.1128/jb.177.14.4121-4130.1995 PMC177145

[CR17] Hammarström M, Woestenenk EA, Hellgren N et al (2006) Effect of N-terminal solubility enhancing fusion proteins on yield of purified target protein. J Struct Funct Genomics 7:1–14. 10.1007/s10969-005-9003-7 16850178 10.1007/s10969-005-9003-7

[CR18] Hammer Ø, Harper D, Ryan P (2001) Past: paleontological statistical software package for education and data analysis. Palaeontologia Electronica 4:1–9

[CR19] Jakab G, Manrique A, Zimmerli L et al (2003) Molecular characterization of a novel lipase-like pathogen-inducible gene family of *Arabidopsis*. Plant Physiol 132:2230–2239. 10.1104/pp.103.025312 12913177 10.1104/pp.103.025312 PMC181306

[CR20] Jevševar S, Gaberc-Porekar V, Fonda I et al (2005) Production of nonclassical inclusion bodies from which correctly folded protein can be extracted. Biotechnol Prog 21:632–639. 10.1021/BP0497839 15801811 10.1021/bp0497839

[CR21] Jin S, Wu Q, Fu G et al (2025) Breaking Evolution’s Ceiling: AI-Powered Protein Engineering. Catalysts 15:842. 10.3390/catal15090842

[CR22] Kaur J, Kumar A, Kaur J (2018) Strategies for optimization of heterologous protein expression in *E. coli*: Roadblocks and reinforcements. Int J Biol Macromol 106:803–822. 10.1016/j.ijbiomac.2017.08.080 28830778 10.1016/j.ijbiomac.2017.08.080

[CR23] Khan MF, Khan MT (2026) AI-Driven Enzyme Engineering: Emerging Models and Next-Generation Biotechnological Applications. Molecules 31 10.3390/molecules31010045 PMC12786422 41515342

[CR24] Kumar J, Chauhan AS, Shah RL et al (2020) Amino acid supplementation for enhancing recombinant protein production in *E. coli*. Biotechnol Bioeng 117:2420–2433. 10.1002/BIT.27371 32369182 10.1002/bit.27371

[CR25] Laemmli UK (1970) Cleavage of Structural Proteins during the Assembly of the Head of Bacteriophage T4. Nature 227:680–685. 10.1038/227680a0 5432063 10.1038/227680a0

[CR26] Lozano Terol G, Gallego-Jara J, Sola Martínez RA et al (2021) Impact of the expression system on recombinant protein production in *Escherichia coli* BL21. Front Microbiol 12:682001. 10.3389/FMICB.2021.682001/BIBTEX 34234760 10.3389/fmicb.2021.682001 PMC8257044

[CR27] Mamipour M, Yousefi M, Hasanzadeh M (2017) An overview on molecular chaperones enhancing solubility of expressed recombinant proteins with correct folding. Int J Biol Macromol 102:367. 10.1016/J.IJBIOMAC.2017.04.025 28412337 10.1016/j.ijbiomac.2017.04.025 PMC7185796

[CR28] Mühlmann M, Forsten E, Noack S, Büchs J (2017) Optimizing recombinant protein expression via automated induction profiling in microtiter plates at different temperatures. Microb Cell Fact 16:1–12. 10.1186/S12934-017-0832-4/FIGURES/5 29183374 10.1186/s12934-017-0832-4 PMC5706349

[CR29] Ormö M, Cubitt AB, Kallio K et al (1996) Crystal structure of the Aequorea victoria green fluorescent protein. Science 273:1392–1395. 10.1126/SCIENCE.273.5280.1392 8703075 10.1126/science.273.5280.1392

[CR30] Ramón A, Señorale-Pose M, Marín M (2014) Inclusion bodies: Not that. bad… Front Microbiol 5:2010–2015. 10.3389/fmicb.2014.00056 10.3389/fmicb.2014.00056 PMC3924032 24592259

[CR31] Remington SJ (2011) Green fluorescent protein: A perspective. Protein Sci 20:1509–1519. 10.1002/pro.684 21714025 10.1002/pro.684 PMC3190146

[CR32] Rosano GL, Morales ES, Ceccarelli EA (2019) New tools for recombinant protein production in *Escherichia coli*: A 5-year update. Protein Sci 28:1412–1422. 10.1002/PRO.3668 31219641 10.1002/pro.3668 PMC6635841

[CR33] Sambrook J, Fritsch EF, Maniatis T (1989) Molecular cloning: a laboratory manual

[CR34] San-Miguel T, Pérez-Bermúdez P, Gavidia I (2013) Production of soluble eukaryotic recombinant proteins in *E. coli* is favoured in early log-phase cultures induced at low temperature. Springerplus 2:1–4. 10.1186/2193-1801-2-89 23525091 10.1186/2193-1801-2-89 PMC3602615

[CR35] Schleif R (2010) AraC protein, regulation of the l-arabinose operon in *Escherichia coli*, and the light switch mechanism of AraC action. FEMS Microbiol Rev 34:779–796. 10.1111/J.1574-6976.2010.00226.X 20491933 10.1111/j.1574-6976.2010.00226.x

[CR36] Shirai K, Jackson RL (1982) Lipoprotein lipase-catalyzed hydrolysis of p-nitrophenyl butyrate. Interfacial activation by phospholipid vesicles. J Biol Chem 257:1253–1258. 10.1016/s0021-9258(19)68183-4 6895751

[CR37] Singh SM, Upadhyay AK, Panda AK (2008) Solubilization at high pH results in improved recovery of proteins from inclusion bodies of *E. coli*. J Chem Technol Biotechnol 83:1126–1134. 10.1002/jctb.1945

[CR38] Slouka C, Kopp J, Strohmer D et al (2019) Monitoring and control strategies for inclusion body production in *E. coli* based on glycerol consumption. J Biotechnol 296:75–82. 10.1016/J.JBIOTEC.2019.03.014 30904592 10.1016/j.jbiotec.2019.03.014

[CR39] Sørensen HP, Mortensen KK (2005) Advanced genetic strategies for recombinant protein expression in *Escherichia coli*. J Biotechnol 115:113–128. 10.1016/j.jbiotec.2004.08.004 15607230 10.1016/j.jbiotec.2004.08.004

[CR40] Upadhyay V, Singh A, Jha D et al (2016) Recovery of bioactive protein from bacterial inclusion bodies using trifluoroethanol as solubilization agent. Microb Cell Fact 15:1–13. 10.1186/s12934-016-0504-9 27277580 10.1186/s12934-016-0504-9 PMC4898390

[CR41] van der Kant R, van Durme J, Rousseau F, Schymkowitz J (2019) Solubis: optimizing protein solubility by minimal point mutations. Methods in Molecular Biology. Humana Press Inc., pp 317–333 10.1007/978-1-4939-8820-4_21 30341620

[CR42] Ventura S, Villaverde A (2006) Protein quality in bacterial inclusion bodies. Trends Biotechnol 24:179–185. 10.1016/J.TIBTECH.2006.02.007 16503059 10.1016/j.tibtech.2006.02.007

[CR43] Waldo GS, Standish BM, Berendzen J, Terwilliger TC (1999) Rapid protein-folding assay using green fluorescent protein. Nat Biotechnol 17:691–695. 10.1038/10904 10404163 10.1038/10904

[CR44] Wang L (2009) Towards revealing the structure of bacterial inclusion bodies. Prion 3:139–145. 10.4161/PRI.3.3.9922 19806034 10.4161/pri.3.3.9922 PMC2802778

