# Supplementary material for: Recombinant expression of natural Arabidopsis PRLIP1 variants reveals temperature-dependent differences in fluorescence and functional protein recovery in Escherichia coli
Source: World J Microbiol Biotechnol. 2026 Jul 10;42(8):406. doi: 10.1007/s11274-026-05138-y (PMC13354648; doi:10.1007/s11274-026-05138-y)
Supplement: Supplementary file 1 — Supplementary Material 1 [file 11274_2026_5138_MOESM1_ESM.docx]

**Supplementary Information**

**Major, Kovács & Jakab
Recombinant expression of natural *Arabidopsis* PRLIP1 variants reveals temperature-dependent differences in fluorescence and functional protein recovery in *Escherichia coli***

| **Gene** | **Primers** | **Sequence** |
| --- | --- | --- |
| PRLIP1 | Reverse | 5’-ccactagccaccctcgaaatc-3’ |
|  | Forward | 5’-cgttacgtgattgcgttacg-3’ |

**Supplementary Table 1. List of primers used in the study.**

| **Temperature (°C)** | **Pearson's r** | **n** | **p-value** |
| --- | --- | --- | --- |
| 37 | -0.01937 | 10 | 0.957641377 |
| 22 | 0.97425 | 10 | 1.86459E-06 |
| 15 | 0.72611 | 9 | 0.0267542 |

**Supplementary Table 2. Temperature-specific correlation between GFP fluorescence and esterase activity.**

| **Variant** | **Cultivation temperature (°C)** | **Esterase activity normalized to total protein (nU µg⁻¹ total protein)** | **Relative GFP-PRLIP1 abundance in IB (37 °C = 1.00)** | **Relative target-corrected recovered activity (a.u.)** | **Relative corrected activity vs 37 °C** |
| --- | --- | --- | --- | --- | --- |
| GFP-ColLip1 | 37 | 0.208 | 1 | 0.208 | 1 |
| GFP-ColLip1 | 22 | 0.502 | 1.216 | 0.413 | 1.99 |
| GFP-ColLip1 | 15 | 0.869 | 0.835 | 1.04 | 5 |
| GFP-WsLip1 | 37 | 0.155 | 1 | 0.155 | 1 |
| GFP-WsLip1 | 22 | 0.619 | 1.787 | 0.346 | 2.24 |
| GFP-WsLip1 | 15 | 1.218 | 1.909 | 0.638 | 4.12 |

**Supplementary Table 3. Relative correction of recovered esterase activity for GFP-PRLIP1 target protein abundance in inclusion body fractions**. Esterase activity was measured using p-nitrophenyl butyrate after alkaline solubilization and dilution-mediated refolding of inclusion body-derived protein preparations. Activity values were normalized to total protein concentration and are expressed as nU µg⁻¹ total protein. Relative GFP-PRLIP1 abundance in the inclusion body fraction was estimated by densitometric analysis of Coomassie-stained SDS-PAGE gels loaded with equal total protein amounts. Band intensities were normalized to the 37 °C inclusion body fraction of the corresponding variant, which was set to 1.00. The target-abundance-corrected recovered activity index was calculated by dividing total-protein-normalized esterase activity by the relative GFP-PRLIP1 band abundance. Because the densitometric values represent relative, not absolute, target protein abundance, the corrected values should be interpreted as relative recovered activity corrected for GFP-PRLIP1 abundance, not as absolute catalytic efficiency.

**(a)**

**catggcatggatgagctcggtaccccggggatccacgaaaattggaaggaggagctgatgggaaacttaaaa**

**H G M D E L G T P G I H E N W K E E L M G N L K**

**(b)**

**PRLIP1Ws MGNLKKSTRSDELSRSGPPQIPNPDWNNLYHRTTVASCLVQGVYAKERDRENNRNGSESL**

**PRLIP1Col MGNLKQSTRSDELSRSGPPQIPNPDWNNLYHRTTVASYLVQGVYAKERDRENNRNGSESL**

*******:*******************************.************************

**PRLIP1Ws ATPWWKSFNFTLDESEILYDAFDGSIYGAVFQNMINYENTPNSIVVPPRYVIALRGTVPS**

**PRLIP1Col ATPWWKSFNFTLDESEILYDAFDDSIYGAVFQNMINYENTPDSIVVPPRYVIALRGTVPS**

*************************.*****************:********************

**PRLIP1Ws DVSDWIHNSRIVLEKLHGGGKHMHVIRKIYSLVAKHGNTAVWIAGHSLGAGLALLAGKDM**

**PRLIP1Col DVSDWIRNSRIVFEKLHGGGKHMHVIRKIYSLVAKHGNTAVWIAGHSLGAGLALLAGKDM**

********:*****:*************************************************

**PRLIP1Ws AMSGLPVEAYIFNPPISLIPLEQCGYNHELNFVYRLTRDLFKAGIAKVVDLDEGQEGPRY**

**PRLIP1Col AMSGLPVEAYIFNPPISLIPLEQCGYNHKLNCVYRLTRDLFKAGIAKVLDLDEGREGPRY**

******************************:**.****************:*****:*******

**PRLIP1Ws KNLASWRPHLFVNQSDVICSEYIGYFNHVVTMTEAGLGEISRLASGYSVRRMLFGDGENW**

**PRLIP1Col KNLASWRPHLFVNQSDVICSEYIGYFNHVATMTEAGLGEISRLASGYSVRRMLFGDGVNW**

*******************************.***************************.****

**PRLIP1Ws SSSSTPDHLHFLPSAFMIVNKTEASEFYNKHGIHQWWNHMLKQSTTFSPY**

**PRLIP1Col SSSSTPDHLHFLPSAFMIVNKTEASEFYNKHGIHQWWNHMLKQSTTFSSY**

**************************************************.***

**Supplementary Figure 1.**

**(a) Sequence of the linker region between GFP and PRLIP1.** The C-terminal region of GFP is highlighted in green, the N-terminal region of PRLIP1 is highlighted in yellow, and the BamHI restriction site within the linker is highlighted in turquoise.

**(b) Sequence comparison of PRLIP1 protein variants from *Arabidopsis* *thaliana* Columbia (PRLIP1Col) and Wassilewskija (PRLIP1Ws) accessions.**

Asterisks (*) represent when two amino acids are identical, periods (.) represent semi-conserved substitutions, and colons (:) represent conserved substitutions.

**
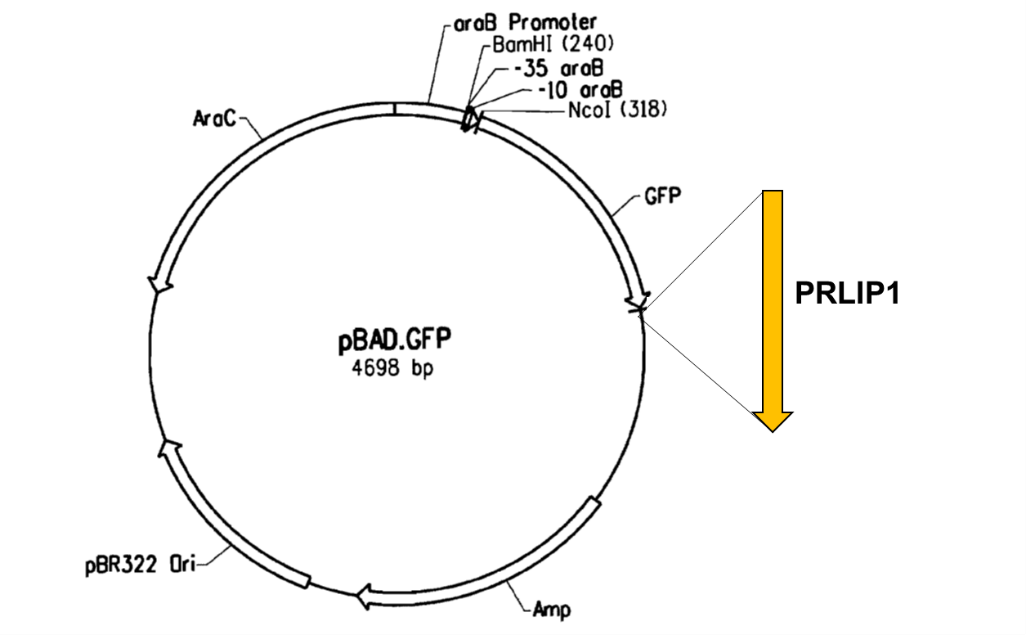
**

**Supplementary Figure 2.** **Construction of pBAD-GFP-PRLIP1 expression vectors.**
Schematic representation of the pBAD-based constructs used for heterologous expression of PRLIP1 fused downstream of GFP.


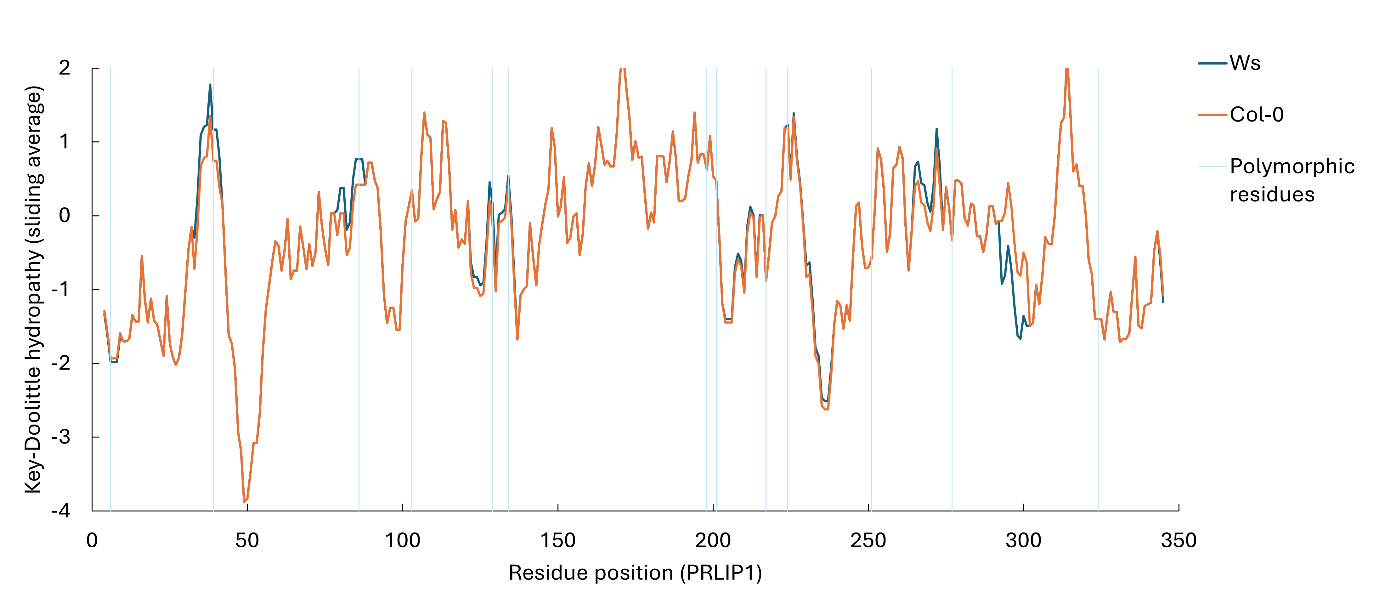


**Supplementary Figure 3.** Kyte–Doolittle hydropathy analysis of PRLIP1 variants using a nine-residue sliding window. Hydrophobicity scores are plotted against residue position for the Columbia-0 (Col-0) and Wassilewskija (Ws) sequences.


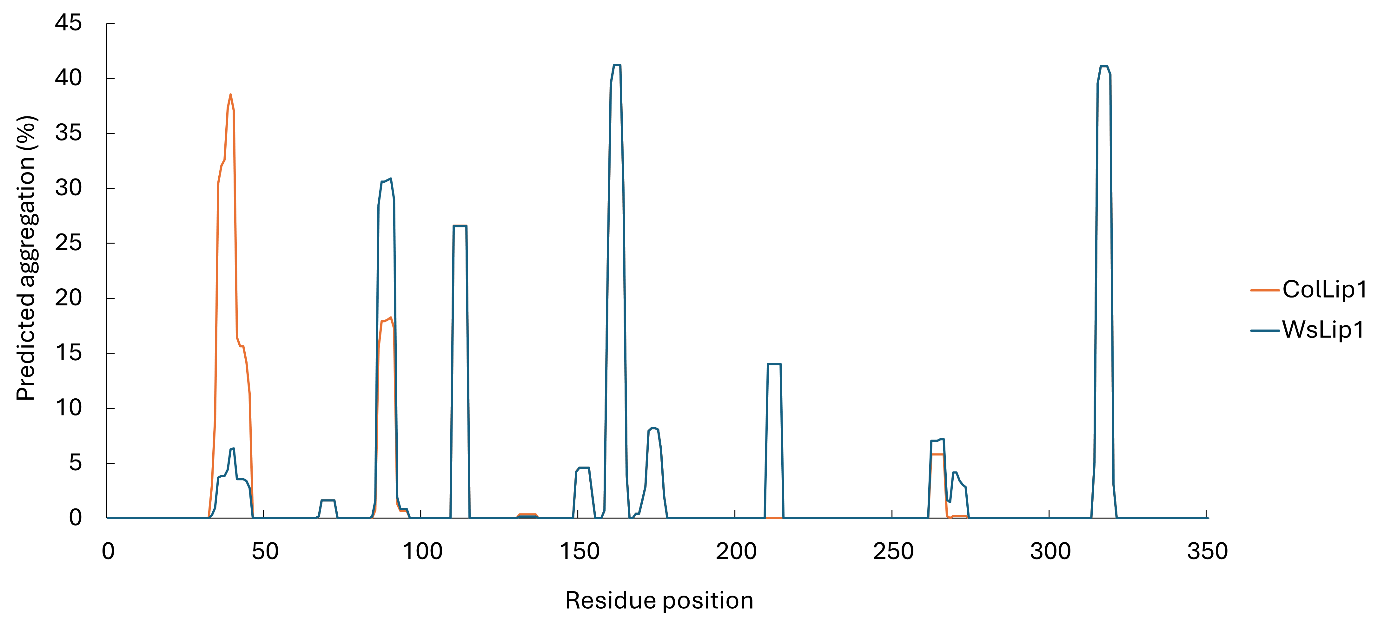


**Supplementary Figure 4**. Per-residue β-aggregation propensity of PRLIP1 variants predicted using the TANGO algorithm under standard conditions (pH 7.0, 298 K, ionic strength 0.15 M). The Columbia-0 (Col-0) and Wassilewskija (Ws) sequences of the PRLIP1 protein were analysed using identical parameters, and aggregation scores are plotted as a function of residue position.


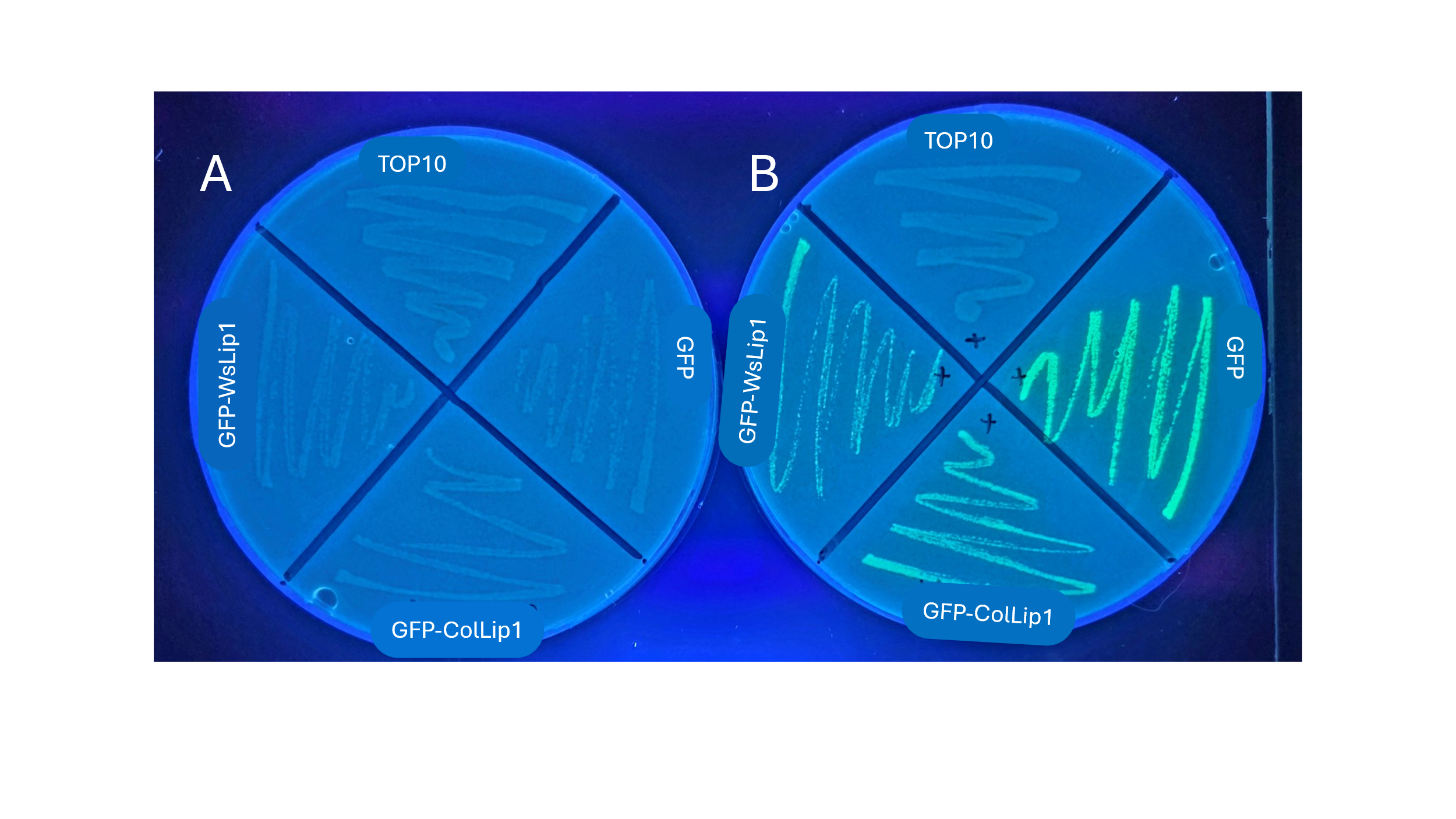


**Supplementary Figure 5. Comparison of fluorescence phenotypes in *E. coli* TOP10 and recombinant strains expressing GFP fusion proteins grown on selective agar plates at 22°C.**

**(a)** Fluorescence of uninduced cultures grown at 22 °C on LB–agar plates containing 100 µg/mL ampicillin. Shown are untransformed TOP10 cells and TOP10 cells harbouring plasmids expressing GFP, GFP-ColLip1, or GFP-WsLip1, all grown in the absence of L-arabinose.

**(b)** Fluorescence of cultures grown under identical conditions but induced with L-arabinose to express recombinant GFP, GFP-ColLip1, or GFP-WsLip1 proteins. Induction reveals distinct fluorescence intensities corresponding to the different fusion constructs.


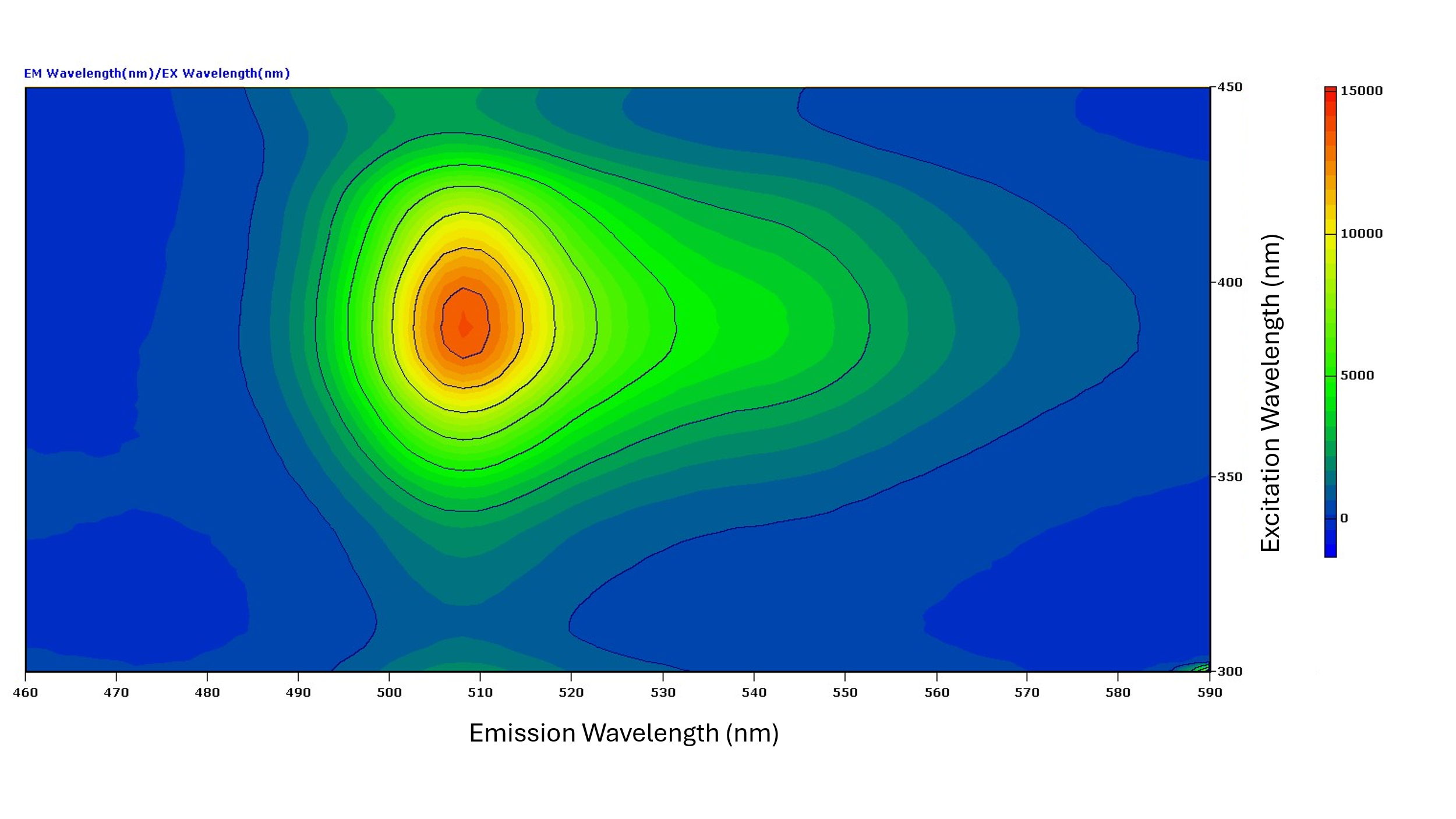


**a**


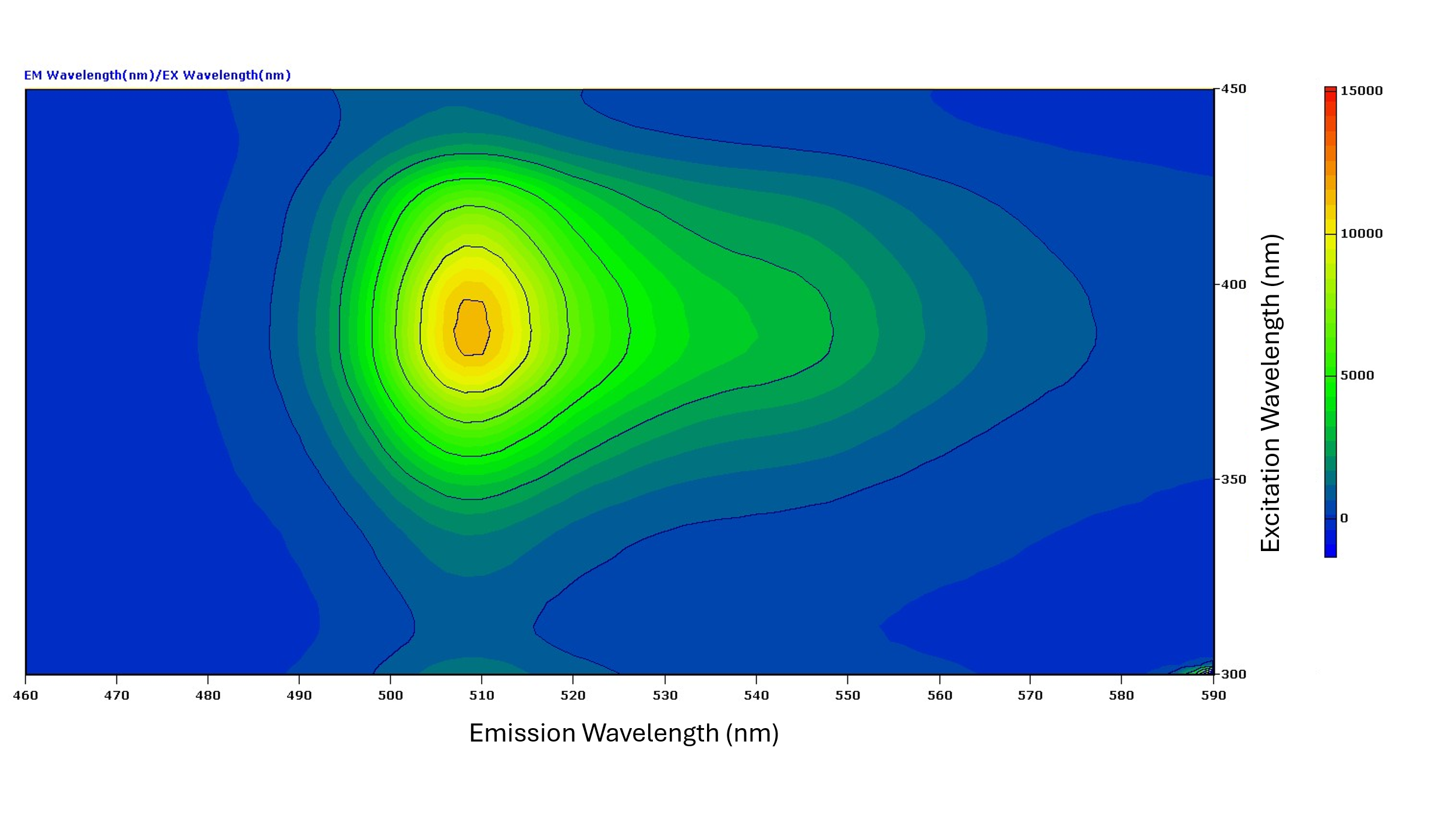


**b**

**Supplementary Figure 6.** **Emission matrices of E. coli strains expressing different vector constructs under identical conditions.**
**(a)** pBAD-GFP (GFP-expressing control).
**(b)** pBAD-GFP-PRLIP1.
Emission profiles were recorded to assess whether PRLIP1 fusion affects GFP fluorescence properties. The bacteria were cultured at 22°C.


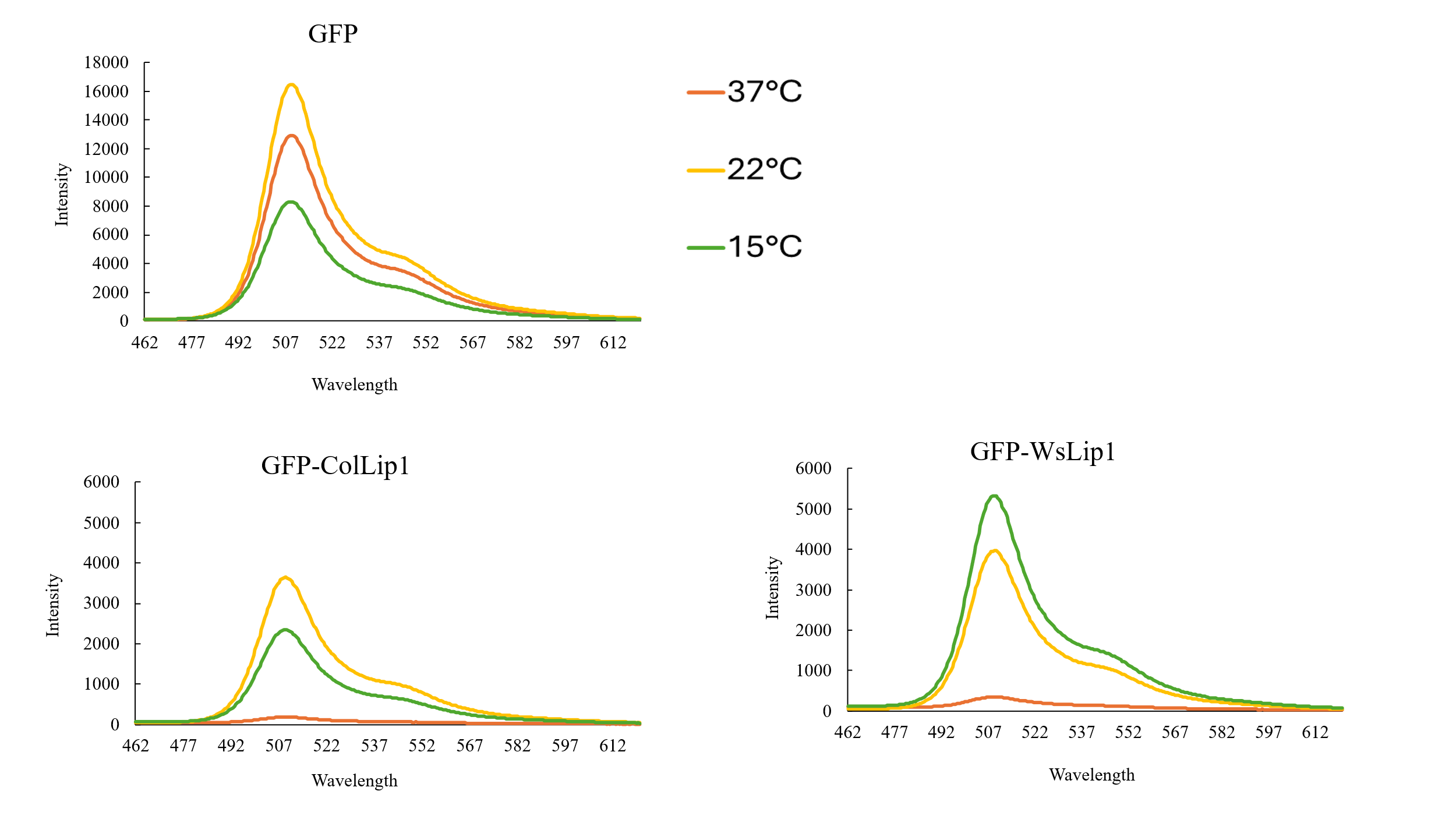


**Supplementary Figure 7**. **GFP fluorescence intensity of induced E. coli cells expressing either GFP alone or GFP-PRLIP1 fusion proteins cultured at different temperatures.**
Whole-cell fluorescence was measured to compare expression levels and solubility across conditions.


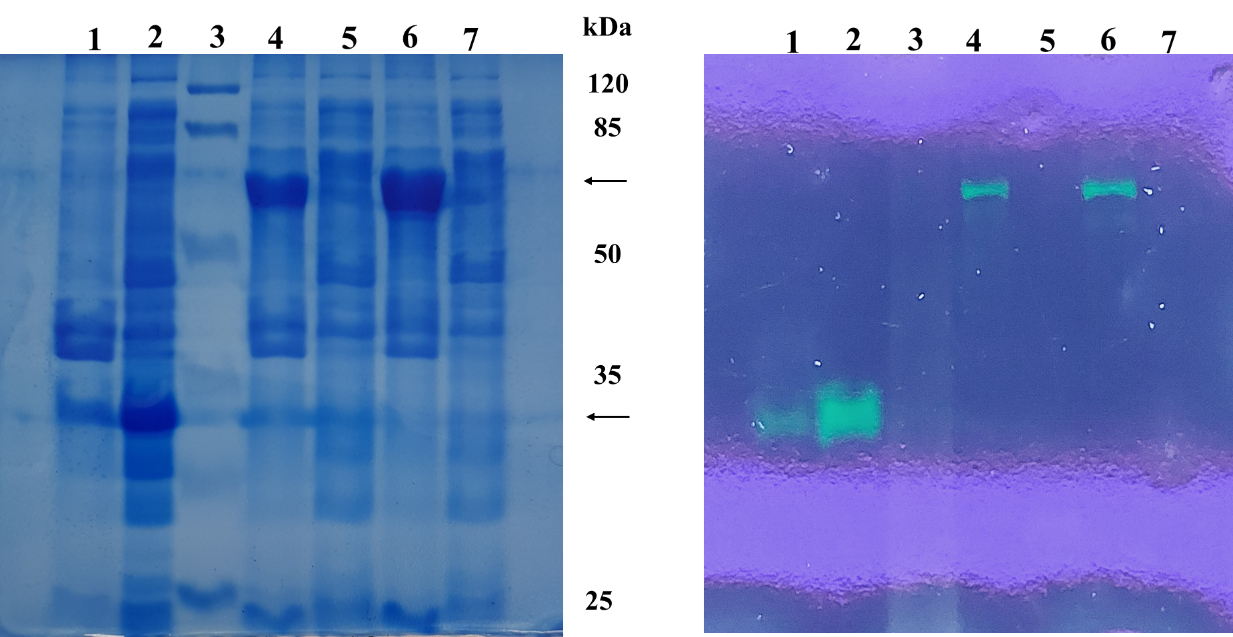


**Supplementary Figure 8.** **Comparison of recombinant protein expression and GFP fluorescence by SDS-PAGE analysis.**

Coomassie Brilliant Blue-stained SDS-PAGE and in-gel fluorescence images showing recombinant protein localization. Arrows indicate the expected molecular weights: GFP-PRLIP1 (~64 kDa) and GFP (~27 kDa), respectively. The bacterial culture was performed at 37°C for 4 hours. After induction of protein expression, the growth temperature was reduced to 22°C for 48 hours. Lane 1: inclusion body, pBAD-GFP; Lane 2: supernatant, pBAD-GFP; Lane 3: molecular weight marker; Lane 4: inclusion body, pBAD-GFP-ColLip1; Lane 5: supernatant, pBAD-GFP-ColLip1; Lane 6: inclusion body, pBAD-GFP-WsLip1; Lane 7: supernatant, pBAD-GFP-WsLip1.


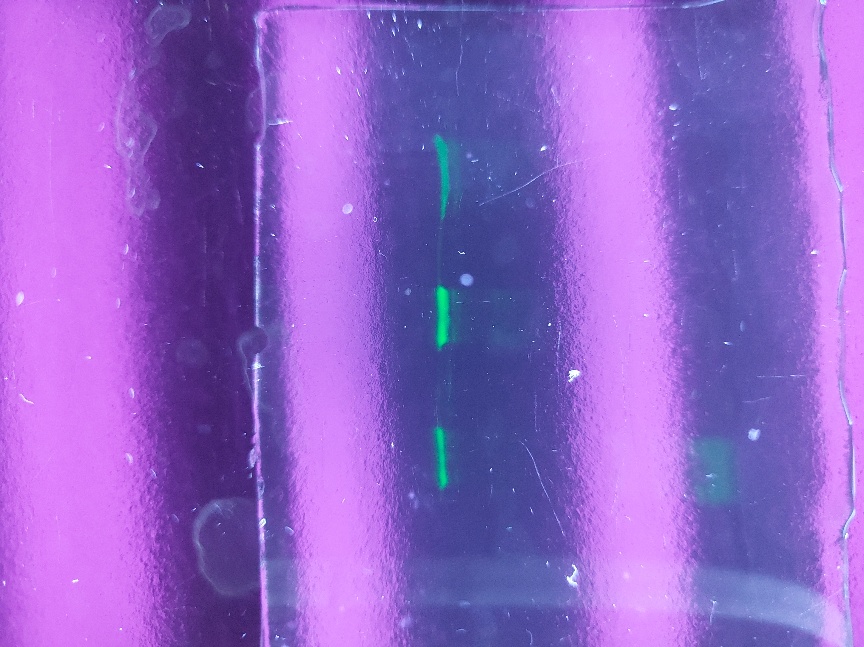


1 2 3 4 5 6 MW


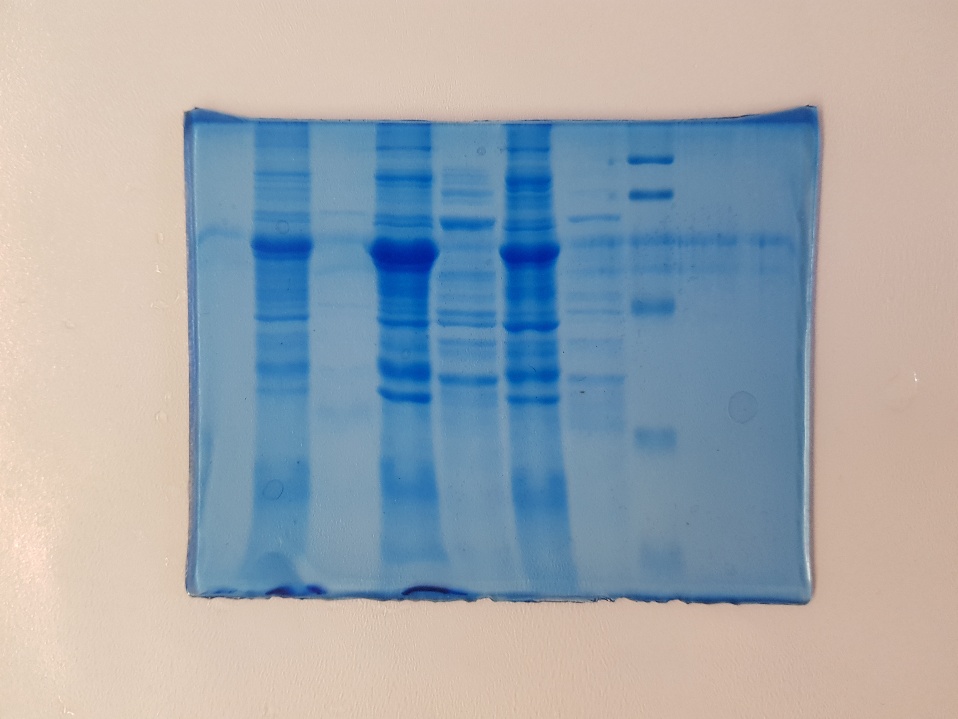


kDa
 120 85

50

35

25

1 2 3 4 5 6 MW

**Supplementary Figure 9. Original uncropped SDS-PAGE gels corresponding to Fig. 2a.**

Uncropped images of the SDS–PAGE gels used to generate the cropped panels shown in Fig. 2a, analysing recombinant GFP-PRLIP1 (WsLip1) expression in *Escherichia coli* grown at different culture temperatures. The gels show in-gel GFP fluorescence (upper panel) and the corresponding Coomassie Brilliant Blue–stained SDS–PAGE gel (lower panel). The GFP-PRLIP1 fusion protein migrates at approximately 64 kDa. Lane assignments are identical to those described in Fig. 2a: Lane 1: inclusion body (37°C); Lane 2: supernatant (37°C); Lane 3: inclusion body (22°C); Lane 4: supernatant (22°C); Lane 5: inclusion body (15°C); Lane 6: supernatant (15°C); Lane 7: molecular weight marker (PageRuler™ Unstained Protein Ladder). The regions shown in the main figure were cropped from these original images for clarity. Molecular weight markers and gel edges are visible in the uncropped images.


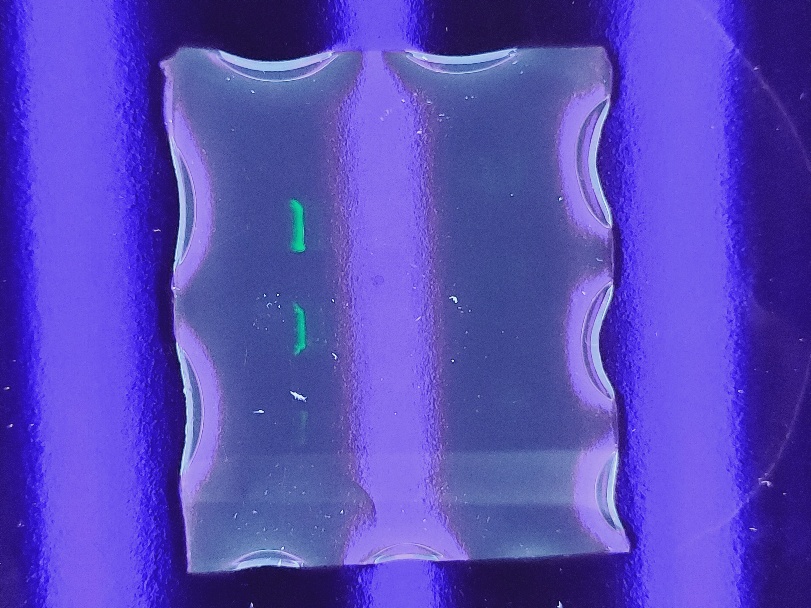


MW 2 3 4 5 6 7


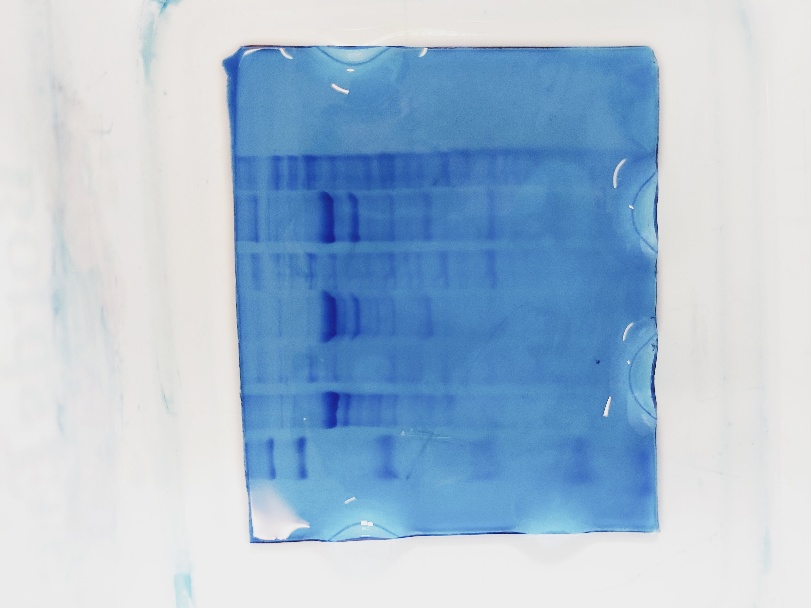


kDa
 120 85

50

35

25

MW 2 3 4 5 6 7

**Supplementary Figure 10. Original uncropped SDS-PAGE gels corresponding to Fig. 3a.**

Uncropped images of the SDS–PAGE gels used to generate the cropped panels shown in Fig. 3a, analysing recombinant GFP-PRLIP1 (ColLip1) expression in *Escherichia coli* cultivated at different temperatures. The gels show in-gel GFP fluorescence (upper panel) and the corresponding Coomassie Brilliant Blue–stained SDS–PAGE gel (lower panel). The GFP-PRLIP1 fusion protein migrates at approximately 64 kDa. Lane assignments are identical to those described in Fig. 3a: Lane 1, molecular weight marker (PageRuler™ Unstained Protein Ladder); Lane 2, inclusion body fraction (37°C); Lane 3, soluble supernatant fraction (37°C); Lane 4, inclusion body fraction (22°C); Lane 5, soluble supernatant fraction (22°C); Lane 6, inclusion body fraction (15°C); Lane 7, soluble supernatant fraction (15°C). The regions presented in the main figure were cropped from these original images to improve clarity. Molecular weight markers and gel edges are visible in the uncropped images.
